# Discovery of VH domains that allosterically inhibit ENPP1

**DOI:** 10.1038/s41589-023-01368-5

**Published:** 2023-07-03

**Authors:** Paige E. Solomon, Colton J. Bracken, Jacqueline A. Carozza, Haoqing Wang, Elizabeth P. Young, Alon Wellner, Chang C. Liu, E. Alejandro Sweet-Cordero, Lingyin Li, James A. Wells

**Affiliations:** 1 Department of Pharmaceutical Chemistry, University of California San Francisco, San Francisco, CA USA; 2 Department of Biochemistry, Stanford University Medical School, Stanford, CA USA; 3 Sarafan ChEM-H, Stanford University, Stanford, CA USA; 4 Macromolecular Structural Knowledge Center, Stanford University, Stanford, CA USA; 5 Division of Pediatric Oncology, Department of Pediatrics, University of California San Francisco, San Francisco, CA USA; 6 Department of Biomedical Engineering, University of California, Irvine, CA USA; 7 Department of Chemistry, University of California, Irvine, CA USA; 8 Department of Molecular Biology and Biochemistry, University of California, Irvine, CA USA; 9 Department of Cellular & Molecular Pharmacology, University of California San Francisco, San Francisco, CA USA; 10 Present Address: Cartography Biosciences, South San Francisco, CA USA

**Keywords:** Protein design, Cancer therapy, Immunology, Enzymes

## Abstract

Ectodomain phosphatase/phosphodiesterase-1 (ENPP1) is overexpressed on cancer cells and functions as an innate immune checkpoint by hydrolyzing extracellular cyclic guanosine monophosphate adenosine monophosphate (cGAMP). Biologic inhibitors have not yet been reported and could have substantial therapeutic advantages over current small molecules because they can be recombinantly engineered into multifunctional formats and immunotherapies. Here we used phage and yeast display coupled with in cellulo evolution to generate variable heavy (VH) single-domain antibodies against ENPP1 and discovered a VH domain that allosterically inhibited the hydrolysis of cGAMP and adenosine triphosphate (ATP). We solved a 3.2 Å-resolution cryo-electron microscopy structure for the VH inhibitor complexed with ENPP1 that confirmed its new allosteric binding pose. Finally, we engineered the VH domain into multispecific formats and immunotherapies, including a bispecific fusion with an anti-PD-L1 checkpoint inhibitor that showed potent cellular activity.

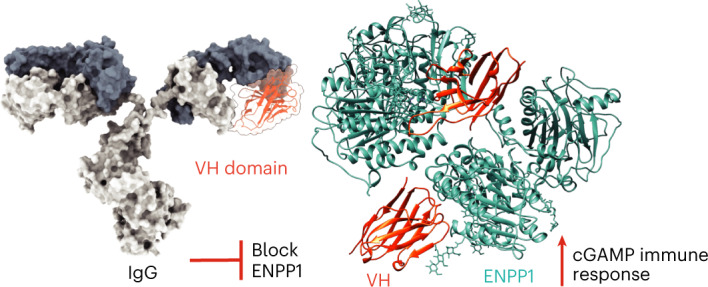

## Abstract (web summary)

Phage and yeast display were used to generate variable heavy single-domain antibodies that bind and allosterically inhibit ectodomain phosphatase/phosphodiesterase-1 to increase the half-life of the immune-stimulant cyclic guanosine monophosphate adenosine monophosphate (cGAMP) in tumor microenvironments.

## Main

Ectodomain phosphatase/phosphodiesterase-1 (ENPP1) is an extracellular enzyme that promotes immune evasion in the context of cancer progression and metastasis. ENPP1 is an immune checkpoint that modulates innate and adaptive immune pathways by hydrolyzing extracellular cyclic guanosine monophosphate adenosine monophosphate (cGAMP) and adenosine triphosphate (ATP)^1–4^. cGAMP is produced by cyclic GMP–AMP synthase in response to the detection of nucleic acid in the cytosol, which occurs after viral infection, and also in tumor cells. Cells export and import cGAMP, triggering intercellular activation of type 1 interferons that coordinate a pleiotropic immune response and recruitment of immune cells^1,3–7^. Simultaneously, cGAMP acts as a negative regulator of metastasis by suppressing epithelial–mesenchymal transition pathways in tumor cells and promoting antitumor responses in immune cells^4–6,8,9^. The tumor microenvironment (TME) can have high concentrations of extracellular cGAMP, and ENPP1 is frequently upregulated on tumor cells to evade *trans* cGAMP signaling, potentiating immune evasion and metastasis^1,3–8^.

Accordingly, there is substantial interest in inhibiting ENPP1 to overcome therapeutic challenges for cancers that exclude lymphocytes from the TME (immune ‘cold’ tumors)^1–3,6,10–14^. Immunotherapies such as checkpoint inhibitors that target cytotoxic T-lymphocyte-associated protein 4 (CTLA-4) or PD-1/PD-L1 and ionizing radiation require tumor-infiltrating lymphocytes and are less effective against immune ‘cold’ tumors^15–20^. Small-molecule inhibitors of ENPP1 have set therapeutic precedent for blocking the hydrolysis of endogenous cGAMP in the TME to sensitize tumor cells to immunotherapy and radiation^1–3,6,10–14^. Stimulator of interferon genes and toll-like receptor activators similarly aim to inflame ‘cold’ tumors through type 1 interferon signaling but require direct intratumoral administration to prevent cytokine-mediated toxicities in healthy tissues^21–26^. Extending the half-life of endogenous cGAMP via ENPP1 inhibition is an alternative strategy. However, there is still evidence of hyperactive immune responses when ENPP1 is targeted systemically, underscoring the need to optimize the selectivity of ENPP1 inhibitors for tumor cells^3^.

ENPP1 is expressed and secreted by normal tissues and controls multiple physiological pathways, creating concerns for off-tumor toxicities and obstacles for on-tumor drug delivery^27–29^. ENPP1 metabolizes nucleotide substrates and is a central regulator of purinergic signaling and inorganic phosphate levels^29,30^. Genetic deletions of ENPP1 in mice and variants of ENPP1 discovered in genome-wide association studies have connected ENPP1 activity to musculoskeletal mineralization and cardiovascular calcification diseases^27,30–35^. ENPP1 levels have also been associated with insulin resistance, plasma cell survival, cell motility and premature aging, emphasizing its multifunctional biology^27,30,36–39^. Additionally, ENPP1 on healthy tissues and in plasma sequesters systemically administered inhibitors and interferes with the delivery of drug to tumor cells, affecting therapeutic potency and minimum dose concentrations. Therefore, therapeutic ENPP1 inhibitors require optimal selectivity for tumor cells and ideally would not be competitive with high levels of ATP and cGAMP in the TME, properties that can be difficult to design using a small-molecule approach.

Biologics, such as antibodies, are a class of therapeutics that are ideal for recombinant engineering into multivalent formats and have strong pharmacokinetic profiles. In contrast to small molecules, an antibody-based inhibitor of ENPP1 could be optimized for tumor cell selectivity by reformatting into multivalent constructs that recognize a second tumor-specific antigen. Notably, such antibodies are immune handles for antibody-dependent cellular cytotoxicity (ADCC) responses by effector cells and for T-cell-directed killing when formatted as a T-cell engager. Therefore, an antibody-based inhibitor of ENPP1 could synergize cGAMP activation with targeted immunotherapy to amplify efficacy.

Here we describe the first reported antibody campaign to generate biologics targeting the enzymatic activity of ENPP1. We isolated high-affinity binders to ENPP1 including a variable heavy (VH) domain that allosterically inhibits cGAMP and ATP hydrolysis. We formatted the inhibitor into biparatopic and bispecific molecules with potent cellular activities. Lastly, we solved a 3.2 Å-resolution cryo-electron microscopy (cryo-EM) structure of the VH inhibitor in complex with ENPP1, elucidating the allosteric mechanism of inhibition and providing insights for structure-guided designs of next-generation therapeutics.

## Results

### VH-phage display generated high-affinity binders to ENPP1

**Fig. 1:**
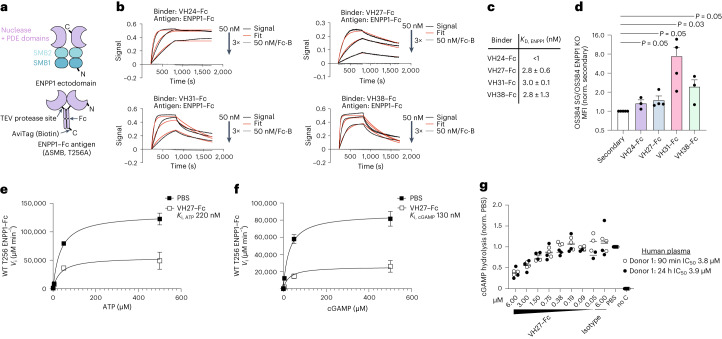
Phage display generated high-affinity VH domains recognizing native ENPP1 on PDX-derived osteosarcoma cells, and VH27–Fc inhibited ATP and cGAMP hydrolysis. **a**, Structure of extracellular domain of ENPP1 and recombinant ENPP1 C-terminal Fc-fusion antigen for phage display (ENPP1–Fc). **b**, Representative biolayer interferometry signals and fits for each VH–Fc binding ENPP1–Fc antigen or Fc-biotin control. **c**, Table of *K*_D_ values (mean for *n* = 2). **d**, VH–Fc binding to PDX-derived OS384 cell line engineered with ENPP1 KO or SG. The bar graph reports mean and s.e.m. of the fold-change (SG/KO) in the median fluorescence intensity (*n* = 3 or 4 independent replicates). Statistics were calculated using a one-tailed Student’s *t* test. **e,f**, Michaelis–Menten kinetics were determined for VH27–Fc using ATP (**e**) or cGAMP (**f**) substrates (mean and s.d. for *n* = 3 independent replicates). **g**, Inhibition of secreted ENPP1 activity in ex vivo human plasma by VH27-Fc. Plasma was supplemented with 1 mM cGAMP and hydrolysis was assayed over 90 min and 24 h time courses. Fc isotype treatment and condition with no cGAMP added (no cGAMP) were included as controls (*n* = 3 for donor 1). Source data

ENPP1 is a type 2 membrane protein with a nuclease-like domain, phosphodiesterase domain and two somatomedin B-like domains (SMB1 and SMB2) orientated toward the extracellular space (Fig. 1a)^40–43^. ENPP1 forms a homodimer on the cell membrane but is also secreted as an enzymatically active monomer^43^. To express antigen for phage display, the ectodomain of human ENPP1 was modified by truncating the SMB1 and SMB2 domains (ΔSMB) and mutating the catalytic threonine to alanine (T256A). For bead-based phage display selections, the C-terminus of the ENPP1 antigen was fused to an Fc domain (ENPP1–Fc) with a C-terminal AviTag via a linker containing a tobacco etch virus (TEV) protease site (Fig. 1a). The biotinylated antigen was immobilized on streptavidin beads for iterative rounds of phage display.

We used our synthetic single-domain VH library based on a stabilized trastuzumab scaffold^44^. VH domains have three complementarity determining regions (CDRs) and generally have weaker affinities than Fabs having six CDRs. However, the smaller size of the VH domain (15 kDa) could have substantial advantages over a Fab (60 kDa) to bind cryptic or sterically challenging sites. Moreover, VH binders can be affinity matured or linked into multivalent formats as a single chain to substantially improve their affinities^44^.

**Extended Data Fig. 1:**
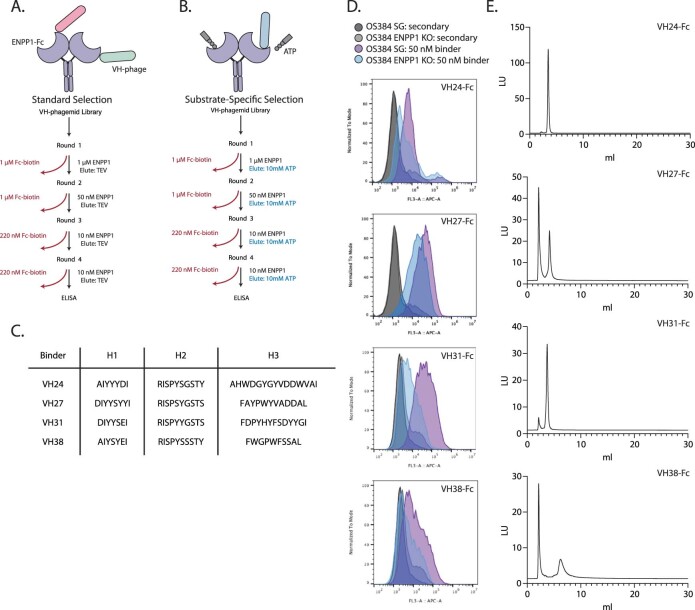
Sequences, cellular binding, and stability of VH panel. **a, b**. Schema of phage display selection and elution by TEV protease treatment **(a)** or 10 mM ATP treatment **(b)**. **c**. CDR H1-3 sequences for VH panel. **d**. Representative flow cytometry histograms used to generate bar graph summarizing fold-changes for binding to OS384 SG and ENPP1 KO in Fig. 1d. **e**. SEC traces for VH24-Fc, VH27-Fc, VH31-Fc, and VH38-Fc.

We performed parallel VH-phage selections using two elution strategies to generate binders globally to ENPP1 and specifically to inhibitory epitopes. In the first standard selection, the bound VH-phage was released using TEV protease treatment to capture all VH binding to ENPP1 ectodomain (Extended Data Fig. 1a). In the second substrate-specific selection, the bound VH-phage was eluted using excess ATP to isolate clones that might compete substrate binding or trap an inactive conformation (Extended Data Fig. 1b). Four rounds of selection were performed by increasing positive and negative selection stringencies as indicated (Extended Data Fig. 1a,b).

The TEV and ATP elution strategies isolated nonoverlapping sets of VH clones that were triaged to a panel of four VH candidates based on affinity and cellular staining (TEV elution, VH24 and VH38; ATP elution, VH27 and VH31; CDR sequences, Extended Data Fig. 1c). First, we expressed bivalent VH–Fc constructs by fusing the C-terminus of the VH domain directly to the Fc hinge. The binding constants of the VH–Fc were measured to be in the single-digit nanomolar range using biolayer interferometry (BLI; Fig. 1b,c). On-cell binding was assayed using a patient-derived xenograft (PDX)-derived osteosarcoma (OS) cell line with high ENPP1 expression (OS384) (refs. ^45,46^). For a paired isogenic comparison, OS384 cells were engineered with CRISPR knockout (KO) of ENPP1 or safe-guide (SG) control. VH–Fc constructs containing VH24, 27, 31 and 38 all recognized epitopes on ENPP1 in its native dimeric form on OS384 cells. VH31–Fc and VH38–Fc were strong on-cell binders with 7.5-fold and 2.5-fold greater staining of SG over ENPP1 KO cell lines. VH24–Fc and VH27–Fc had lower SG over ENPP1 KO staining ratios of 1.3 and 1.5 despite all the binders having similar affinities and dissociation kinetics (Fig. 1d and Extended Data Fig. 1d). It is possible that the epitopes recognized by VH24–Fc and VH27–Fc are less accessible when native ENPP1 is dimerized on the cell membrane compared to the modified ENPP1–Fc antigen. Several factors are the presence of the SMB domains, the orientation of membrane ENPP1 as an N-terminal dimer (versus ENPP1–Fc as a C-terminal dimer) or obstructions from protein interactors on the membrane such as the insulin receptor^36,37^.

Next, we expressed catalytically active (T256) ENPP1–Fc and performed luciferase-based functional assays monitoring ATP or cGAMP hydrolysis to identify VH candidates in the panel that could inhibit ENPP1. VH27–Fc was the only molecule found to functionally inhibit ENPP1–Fc with Michaelis–Menten *K*_i_ values for ATP and cGAMP of 220 nM (95% confidence interval (CI_95_): 160–300 nM) and 130 nM (CI_95_: 97–160 nM), respectively (Fig. 1e,f). Interestingly, *V*_app_ in the presence of VH27–Fc did not reach the *V*_max_ of the enzyme as substrate concentration was increased, indicating that VH27–Fc is noncompetitive with substrate and inhibits ENPP1 by an allosteric mechanism. This was interesting because VH27 was isolated by elution with excess ATP yet was neither directly nor conformationally competitive with the substrate; therefore, its discovery may have been serendipitous.

To confirm that VH27–Fc could also inhibit native soluble ENPP1, we tested the inhibitor in donor human plasma. VH27–Fc inhibited the hydrolysis of cGAMP with IC_50_ values of approximately 3.8 µM and 3.9 µM over 90 min and 24 h, respectively (Fig. 1g). Others have reported small-molecule ENPP1 inhibitors with higher IC_50_ values when tested using plasma or plated cells versus recombinant enzyme^2,12^. The shift in potency reflects nonspecific protein binding in more biologically complex samples. Differences in antigen avidity and epitope accessibility are additional variables that will affect the efficacy of VH27–Fc.

### Yeast display yielded a VH27 variant with improved properties

**Fig. 2:**
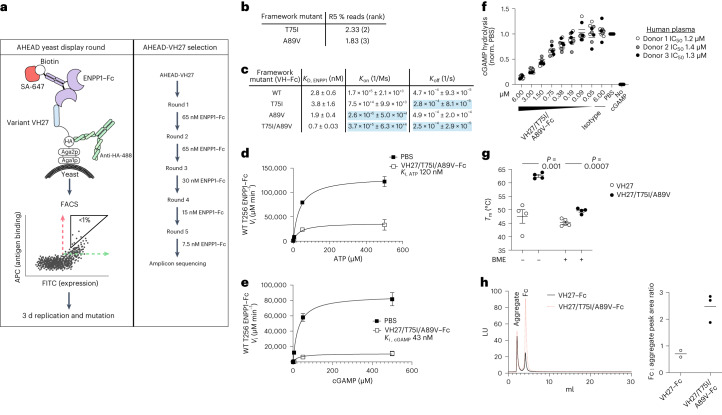
Affinity maturation of VH27 improved affinity, inhibitory potency and stability. **a**, Schema of AHEAD yeast-display selection rounds. **b**, Next-generation sequencing (NGS) read frequencies and ranks for T75I and A89V mutants. **c**, Association and dissociation rates and affinity constants for VH27–Fc, VH27/T75I–Fc, VH27/A89V–Fc and VH27/T75I/A89V–Fc binding to ENPP1–Fc antigen were determined by biolayer interferometry (mean and s.d. for *n* = 2 independent replicates). **d**,**e**, Michaelis–Menten enzyme kinetics for VH27/T75I/A89V–Fc using ATP (**d**) and cGAMP (**e**) substrates (mean and s.d. for *n* = 3 independent replicates). **f**. Inhibition of secreted ENPP1 activity in ex vivo human plasma by VH27/T75I/A89V-Fc (1 mM cGAMP, 90 min). Fc isotype treatment and condition with no cGAMP added (no cGAMP) were included as controls (*n* = 3 for each donor). **g**, Differential scanning fluorimetry was used to measure *T*_m_ of VH27 and VH27/T75I/A89V as single-domain VHs in nonreducing and reducing (6.25% BME) conditions. Bar graph reports mean and s.e.m. for *n* = 4 or 5 independent replicates, and statistics were calculated using two-tailed Student’s *t* test. **h**, VH27–Fc and VH27/T75I/A89V–Fc were analyzed by SEC, and peak area ratios between ‘aggregate peak’ and ‘Fc peak’ were analyzed. Bar graph reports the mean for *n* = 2 or 3 independent replicates. Source data

**Extended Data Fig. 2:**
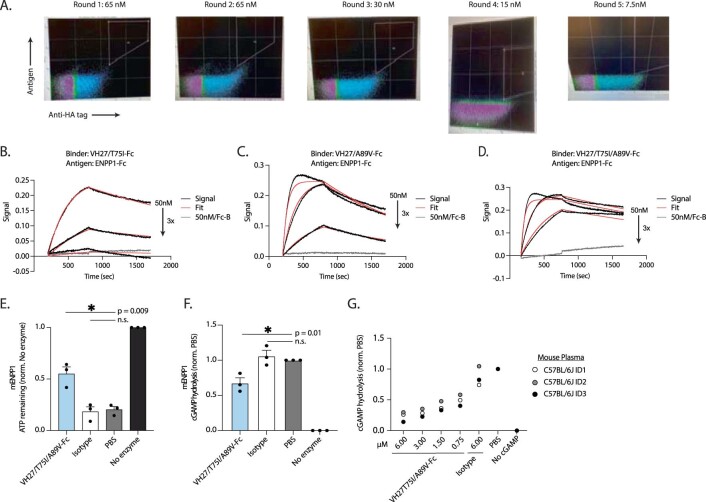
AHEAD yeast display campaign to affinity mature VH27. **a**. FACS gating used for sorting less than 1% AHEAD yeast cell population based on expression (HA-tag) and antigen binding. In round 4 the fluorophores were switched (SA-488 and anti-HA-647). **b–d**. Biolayer interferometry signals and fits to determine K_D_, k_association_, and k_dissociation_ for VH27-Fc with scaffold mutations T75I (**b**), A89V (**c**), and T75I/A89V (**d**). Data are representative of two independent experiments. **e, f**. Mouse ENPP1 (mENPP1) was treated with 500 nM VH27/T75I/A89V-Fc, Fc isotype, or PBS to evaluate cross-reactivity using ATP (**e**) and cGAMP (**f**) substrates. Bar graphs reports mean and SEM for *n* = 3 independent replicates and statistics were calculated using two-tailed Student’s *t* test. **g**. Dose titration of VH27/T75I/A89V-Fc or indicated controls were tested for ENPP1 inhibition in C57BL/6J mouse plasma supplemented with 1 mM cGAMP for 90 min (*n* = 3 mice, 1 experiment per mouse). Source data

Single-chain VH domains are well-suited for affinity maturation to evolve even stronger binders. Having validated VH27 as an ENPP1 inhibitor, we aimed to improve its binding kinetics and stability using autonomous hypermutation yeast surface display (AHEAD)^47^. As previously described, the AHEAD yeast strain uses an orthogonal error-prone polymerase that exclusively replicates and mutagenizes an orthogonal plasmid encoding chosen genes without affecting genomic DNA (OrthoRep system)^47,48^. The AHEAD platform enabled soft-randomization in cellulo, which simplified the cloning compared to the error-prone PCR library preparation and would allow for rediversification should selection bottlenecks occur. We incorporated the *VH27* gene into the plasmid with a hemagglutinin (HA)-tag to quantify expression. In iterative rounds of selection, yeast cells continuously diversifying VH27 sequences were stained with fluorescently labeled antigen and anti-HA antibody and analyzed by fluorescence-activated cell sorting (FACS; Fig. 2a and Extended Data Fig. 2a).

After five rounds of the AHEAD process, several VH27 variants emerged and were detected with next-generation sequencing read abundances over 1%. T75I and A89V were top-ranked mutations located in the VH framework (Fig. 2b). Residue T75I is in a scaffold loop adjacent to the tyrosine-rich CDR H1; this preference for isoleucine could indicate hydrophobic interactions with aromatic CDR H1 residues. The T75I substitution decreased the dissociation rate supporting the hypothesis that it might be extending effects to the CDR configuration (Fig. 2c). Residue A89V is in spatial proximity to the VH domain C-terminus and might affect compaction and stability. The A89V variant enhanced the association rate, which can be related to gains in stability that increase the effective concentration of folded protein (Fig. 2c).

We built a consensus variant (VH27/T75I/A89V–Fc) that had improved association and dissociation rates and a subnanomolar *K*_D_ value (Fig. 2c and Extended Data Fig. 2b–d). These gains corresponded to lower *K*_i_ values for ATP and cGAMP hydrolysis, 120 nM (CI_95_: 83–160 nM) and 43 nM (CI_95_: 30–57 nM), respectively (Fig. 2d,e). VH27/T75I/A89V–Fc was also a threefold stronger inhibitor of cGAMP degradation in plasma with IC_50_ values between 1.2 µM and 1.4 µM for three donors (Fig. 2f). We tested the inhibitor for cross-reactivity with mouse ENPP1 (mENPP1), which has ~80% homology to human ENPP1 (hENPP1)^42^. VH27/T75I/A89V–Fc inhibited the hydrolysis of ATP and cGAMP by recombinant mENPP1 monomer and blocked cGAMP degradation by native mENPP1 in ex vivo C57BL/6J mouse plasma (Extended Data Fig. 2e–g).

**Extended Data Fig. 3:**
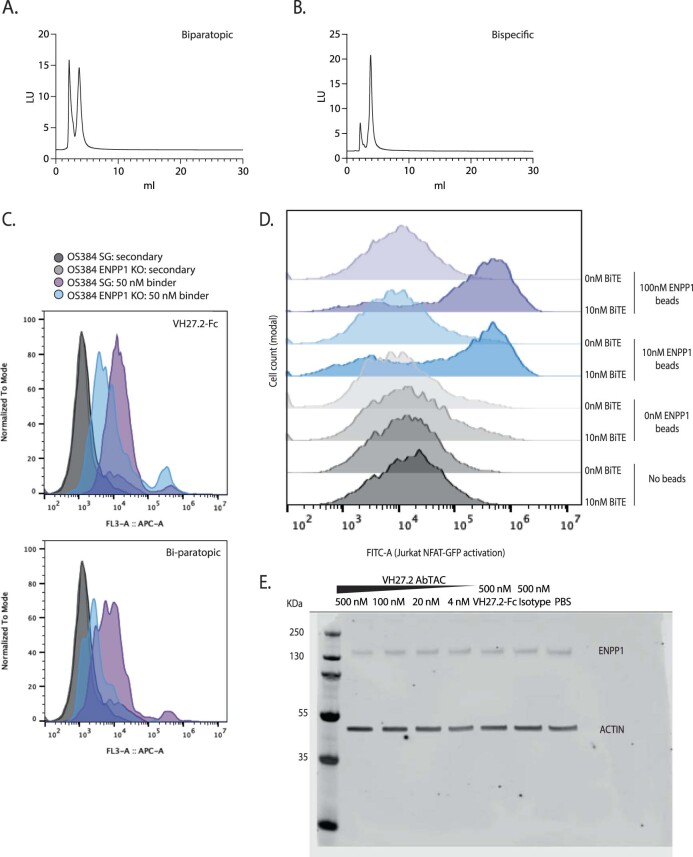
Additional data for multivalent constructs. **a,b**. SEC traces for bi-paratopic inhibitor (**a**) and bispecific inhibitor (**b**). **c**. Representative flow cytometry histograms used to generate bar graph summarizing fold-changes for binding to OS384 SG and ENPP1 KO in Fig. 3f. **d**. Representative flow cytometry histograms for NFAT-GFP Jurkat activation assays when treated with indicated concentrations of ENPP1-coupled beads or no beads and 10 nM BiTE or PBS. **e**. Unformatted immunoblot with visible ladder used for image in Fig. 5f.

Aligned with our hypothesis that these scaffold changes might impact domain stability, the double mutant showed enhanced biophysical properties. Incorporation of the T75I/A89V mutations dramatically increased the thermal melting temperature (*T*_m_) of single-domain VH27 from 48 °C to 63 °C (Fig. 2g). When the VH disulfide was reduced with β-mercaptoethanol (BME), the double mutant was still substantially more stable than VH27, with a *T*_m_ of 50 °C and 45 °C, respectively (Fig. 2g). Finally, the T75I/A89V mutations decreased the level of aggregation observed by size-exclusion chromatography (SEC) when expressed in the VH–Fc format (Fig. 2h). While aggregated VH27–Fc produced some nonspecific cellular stickiness, VH27/T75I/A89V–Fc showed dramatically less background staining on OS384 ENPP1 KO cells (Extended Data Figs. 1d and 3c). We used VH27/T75I/A89V for the rest of the experiments and refer to it as VH27.2 for brevity.

### Multivalent constructs increased cellular inhibition

**Fig. 3:**
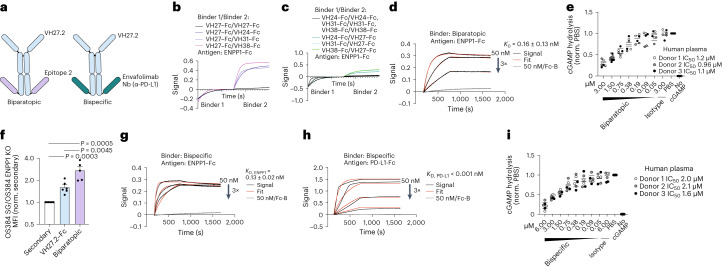
VH27.2 was formatted into biparatopic and bispecific constructs. **a**, Structures of biparatopic and bispecific tetravalent Fc inhibitors. **b**,**c**, Biolayer interferometry mapping the epitopes of the VH panel with respect to the VH27 epitope when VH27–Fc was preloaded on the sensor (**b**), and when VH24–Fc, VH31–Fc or VH38–Fc was preloaded on the sensor (**c**). **d**, Representative biolayer interferometry signals and fits for biparatopic VH27.2/VH31 inhibitor binding ENPP1–Fc antigen or Fc-biotin control (mean and s.d. for *n* = 2 independent replicates). **e**, Inhibition of secreted ENPP1 activity in ex vivo human plasma by biparatopic molecule (1 mM cGAMP, 90 min). Fc isotype treatment and condition with no cGAMP added (no cGAMP) were included as controls (*n* = 2 independent replicates for each donor). **f**, Binding of VH27.2–Fc and biparatopic molecule to OS384 SG and ENPP1 KO cells. The bar graph reports mean and s.e.m. of the fold-change (SG/KO) in the median fluorescence intensity (*n* = 5 or 6 independent replicates). Statistics were calculated using a one-tailed Student’s *t* test. **g**,**h**, Representative biolayer interferometry signals and fits for bispecific inhibitor binding ENPP1–Fc antigen (**g**), PD-L1–Fc antigen (**h**) and Fc-biotin control (mean and s.d. for *n* = 2 independent replicates). **i**. Inhibition of secreted ENPP1 activity in ex vivo human plasma by bispecific molecule (1 mM cGAMP, 90 min). Fc isotype treatment and condition with no cGAMP added (no cGAMP) were included as controls (*n* = 2 independent replicates for each donor). Source data

We tested if the VH inhibitor could be engineered into multivalent formats to enhance potency and tumor specificity. As examples, we built biparatopic and bispecific tetravalent Fc constructs that append a second recognition arm onto the C-terminus of the Fc via a 15 amino acid glycine–serine linker (Fig. 3a). These Fc formats are symmetric and do not require specialized ‘knob-into-hole’ assembly pipelines, could be produced in high yields in mammalian cell culture and are biophysically stable (Extended Data Fig. 3a,b).

To generate a biparatopic inhibitor, we performed epitope binning for the VH–Fc panel and found each could simultaneously engage ENPP1 in the presence of VH27–Fc, indicating the epitopes were nonoverlapping and compatible to combine with VH27–Fc (Fig. 3b,c). We created the VH27.2 biparatopic inhibitor using VH31 because it displayed the best cellular staining of OS384; we hypothesized that the VH31 moiety would increase the local concentration of inhibitor on ENPP1 on the membrane (Fig. 1d). BLI verified that the biparatopic molecule had a subnanomolar binding constant to ENPP1–Fc in vitro, and its IC_50_ in human plasma was measured to be between 0.96 µM and 1.2 µM for three donors (Fig. 3d,e). We stained OS384 SG and ENPP1 KO cells and demonstrated that valency with VH31 increased the specific binding by 69%, with SG over ENPP1 KO ratios of 1.6 and 2.7 for VH27.2–Fc and biparatopic inhibitor, respectively (Fig. 3f and Extended Data Fig. 3c).

Next, we generated a bispecific ENPP1 inhibitor using the clinically approved PD-L1 checkpoint inhibiting nanobody, Envafolimab (Fig. 3a)^49,50^. Several reports have shown that cotreating small-molecule ENPP1 inhibitors with an immune checkpoint blockade substantially enhances tumor regression^11,13,14^. Bispecific biologics combine these two synergizing therapies into a single molecule. Furthermore, overexpressed PD-L1 on various cancers should increase the local concentration of VH27.2 on tumor cell membranes to augment potency and tumor selectivity. We constructed the bispecific VH27.2/Envafolimab inhibitor and confirmed its binding to both ENPP1–Fc and PD-L1–Fc antigens (Fig. 3g,h). The bispecific molecule retained its inhibitory function in human plasma (Fig. 3i).

**Fig. 4:**
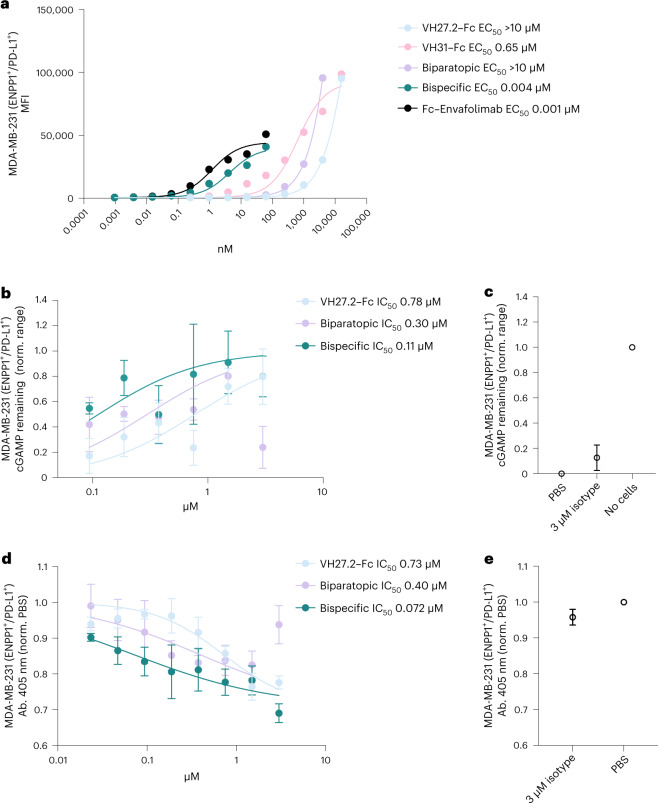
Biparatopic and bispecific constructs improved localization on tumor cells and increased inhibition of ENPP1 on-cell membranes. **a**, MDA-MB-231 cells were stained with a titration of VH27.2–Fc, VH31–Fc, biparatopic molecule, bispecific molecule or Fc–Envafolimab, and median fluorescence intensity values were used to fit EC_50_ curves (mean for *n* = 2 or 3 independent replicates). **b**, MDA-MB-231 cells were treated with cGAMP and the indicated concentration of VH27.2–Fc, biparatopic inhibitor or bispecific inhibitor. cGAMP remaining in the media was measured by cGAMP ELISA. **c**, Data were normalized to the range between the media treated without cells and cells treated with PBS. Fc isotype treated at 3 µM was also included as a control. **d**, MDA-MB-231 cells were treated with pNP-TMP, and the indicated concentration of VH27.2–Fc, biparatopic inhibitor or bispecific inhibitor and pNP-TMP hydrolysis was measured. **e**, Data were normalized to the PBS condition. Fc isotype treated at 3 µM was also included as a control. Data in **b–e** represent mean and s.e.m. for *n* = 3–5 biological replicates. Source data

ENPP1 and PD-L1 are highly expressed on MDA-MB-231 cells; therefore, we used this cell line to measure on-cell binding for the biparatopic molecule compared to VH27.2–Fc and VH31–Fc, as well as for the bispecific molecule relative to VH27.2–Fc and Fc–Envafolimab (Envafolimab fused to the C-terminus of the Fc via a 15 amino acid glycine–serine linker). The EC_50_ values for VH27.2–Fc and VH31–Fc were estimated to be >10 µM and 0.65 µM, respectively, and the biparatopic molecule had a decreased EC_50_ fit relative to VH27.2–Fc (Fig. 4a). While VH27.2–Fc was a weak cellular binder, Fc–Envafolimab robustly stained cells with an EC_50_ value of ~1 nM. The bispecific molecule had an EC_50_ value of ~4 nM and showed nearly equivalent cellular binding as Fc–Envafolimab, indicating that we could effectively leverage PD-L1 dual-recognition to boost inhibitor concentrations on the cell membrane (Fig. 4a).

The inhibitory potencies of the biparatopic and bispecific inhibitors were compared to that of VH27.2–Fc using cellular assays with MDA-MB-231 cells and two substrates of ENPP1, cGAMP (Fig. 4b,c) or p-nitrophenyl thymidine 5′-monophosphate (pNP-TMP; Fig. 4d,e). Although pNP-TMP is a synthetic substrate hydrolyzed by other extracellular phosphodiesterase enzymes, it provides a direct colorimetric read-out enabling higher throughput and lower noise than a cGAMP ELISA. These cellular assays demonstrated equivalent trends for cGAMP and pNP-TMP substrates. VH27.2–Fc inhibited cellular ENPP1 with IC_50_ values between 0.73 µM and 0.78 µM, which was lower than the IC_50_ value for soluble ENPP1 in plasma (Figs. 4b–e and 2f). This is consistent with there being higher avidity for the homodimeric membrane species compared to the secreted monomer, which should increase the ability to specifically target the membrane-bound form of ENPP1.

The biparatopic inhibitor was approximately twice as potent as VH27.2–Fc in cellular assays with an IC_50_ value between 0.30 µM and 0.40 µM (Fig. 4b–e). Biparatopic valency affected the cellular IC_50_ value more dramatically than it changed the plasma IC_50_ value, supporting that the VH31 epitope is highly accessible on membrane-bound ENPP1 and could effectively rescue cellular binding (Figs. 3e and 4b–e). Interestingly, there was a hook effect for the highest concentration of biparatopic molecule in the cell-based assays that was not observed in plasma (Figs. 3e and 4b–e). There may be steric limitations when the VH31 epitope becomes saturated on both subunits of the ENPP1 homodimer.

The bispecific inhibitor, which had orders of magnitude greater cellular localization relative to VH27.2–Fc, correspondingly displayed a sevenfold to tenfold improvement in the cellular IC_50_ values for pNP-TMP and cGAMP substrates, measured to be 0.11 µM and 0.07 µM, respectively (Fig. 4b–e). As observed for the biparatopic inhibitor, there was a distinct improvement in the inhibition of membrane ENPP1 but not the secreted form in plasma (Figs. 3i and 4b–e). This further validated that engineering multispecific recognition of a second tumor epitope or antigen can improve cellular localization to optimize potency. As a result, cotargeting PD-L1 yielded our most effective molecule by driving substantially higher inhibitor concentrations on tumor cell membranes.

### VH domains were engineered into immunotherapy and degrader scaffolds

**Fig. 5:**
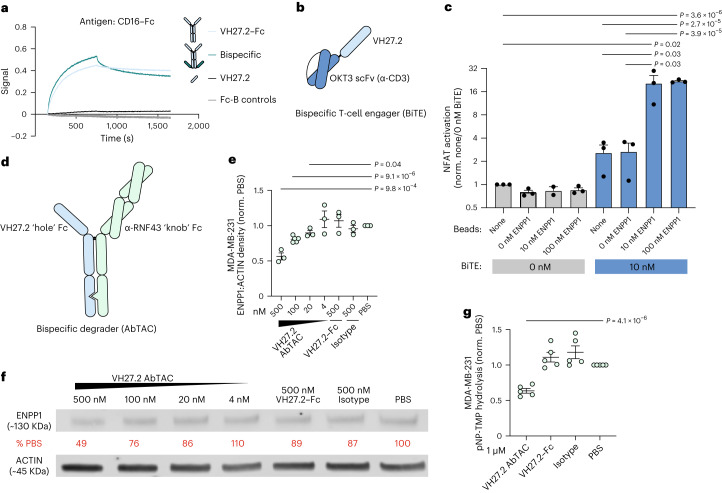
VH27.2 was recombinantly engineered into immunotherapy scaffolds and next-generation protein degraders. **a**, Representative biolayer interferometry signals for bivalent VH27.2–Fc, tetravalent bispecific Fc and single-domain VH27.2 (no Fc) binding to CD16–Fc antigen or Fc-biotin control (*n* = 2). **b**, Structure of bispecific T-cell engager (BiTE) combining VH27.2 with arm recognizing CD3 (OKT3 scFv). **c**, Jurkat cells expressing NFAT–GFP reporter were incubated with beads coated with Fc–ENPP1 or no bead control and were treated with 10 nM BiTE or PBS control. GFP expression driven by NFAT activation was measured by flow cytometry. Data were normalized to the no bead/no BiTE condition. Bar graph reports mean and s.e.m. for *n* = 3 independent replicates. Statistics were calculated using two-tailed Student’s *t* test. **d**, Structure of ‘knob-into-hole’ bispecific AbTAC degrader combining anti-ENPP1 VH with RNF43-recruiting IgG arm. **e**,**f**, MDA-MB-231 cells were treated with PBS or a titration of VH27.2 AbTAC, and ENPP1 levels were measured by immunoblot. Cells were additionally treated with 500 nM VH27.2–Fc and Fc isotype controls. ENPP1 densities were normalized to ACTIN loading control. The percent of ENPP1 remaining relative to the PBS treatment was calculated. **e**, Graph summarizes the mean and s.e.m. for *n* = 3–5 independent replicates. Statistics were calculated using two-tailed Student’s *t* test. **f**, A representative immunoblot for one experiment. **g**, MDA-MB-231 cells were pretreated with VH27.2 AbTAC, indicated control molecules or PBS for 24 h. After removing the molecules and washing the cells, pNP-TMP hydrolysis was measured. Data were normalized to PBS condition, and the graph represents mean and s.e.m. for *n* = 3 independent replicates with 1 or 2 technical replicates. Statistics were calculated using two-tailed Student’s *t* test. Source data

ADCC is a fundamental mechanism of clinically approved therapeutic antibodies^51–53^. In addition to inhibiting ENPP1, constructs containing Fc domains engage Fc receptors such as CD16 on natural killer (NK) cells and macrophages to activate ADCC^54,55^. As expected, the bivalent and tetravalent Fc inhibitors were high-affinity binders of CD16, whereas single-domain VH27.2 did not bind to the Fc receptor (Fig. 5a).

VH domains can be engineered into bispecific T-cell engagers (BiTEs) that incorporate a CD3 recognition arm. We generated a BiTE by linking (15 amino acid glycine–serine linker) VH27.2 with OKT3, a well-established anti-CD3 scFv (Fig. 5b)^56,57^. Jurkat T cells that control the expression of GFP by a nuclear factor of activated T-cells (NFAT) response element (NFAT–GFP reporter) became robustly activated after a 20 h incubation with 10 nM BiTE and ENPP1–Fc immobilized on streptavidin magnetic beads. There was no NFAT response when Jurkat cells were cultured without the BiTE or without ENPP1-coupled beads, demonstrating the high specificity of T-cell activation by the VH27.2 BiTE (Fig. 5c and Extended Data Fig. 3d).

The therapeutic space for biologics and immunotherapies continues to expand, and the VH domain from our panel can be directly ported into new formats. For example, protein degraders are a new class of bispecific molecules with degradation-inducing arms^58–60^. AbTACs incorporate an IgG arm that recruits the membrane-bound E3 ligase RNF43 to ubiquitinate lysine residues on the cytosolic domain of a target protein and designate it for lysosomal degradation^58^. Although only one VH domain in our panel recognized a functional epitope on ENPP1, it is possible that any VH domain can be constructed into an AbTAC to hijack degradation pathways and inactivate ENPP1.

There are three lysine residues in the cytosolic domain of ENPP1 suggesting that it could be a viable target for RNF43 ubiquitination. We constructed ‘knob-into-hole’ bispecific VH–IgG AbTACs for VH24, VH27.2, VH31 and VH38 (Fig. 5d). The VH domains lack a light chain; therefore, complicated steps for light chain pairing were not required. The ‘knob’ and ‘hole’ pieces were co-expressed in a single mammalian transfection and purified using a His tag introduced on the ‘knob’ arm to exclude contaminating ‘hole–hole’ homodimers.

Degradation by the formation of a tertiary complex of E3 ligase, bispecific degrader and target protein is epitope dependent^59,61^. MDA-MB-231 cells, which express RNF43 and have been used for RNF43-mediated degradation of target proteins previously, were treated with AbTACs, and we discovered that the VH27.2 AbTAC was the most potent ENPP1 degrader^58,61^. In total, 44% of ENPP1 was degraded after 24 h incubation with 500 nM VH27.2 AbTAC. Treatment with 500 nM VH27.2–Fc did not decrease ENPP1 levels, validating that the AbTAC is inducing protein degradation as opposed to internalization (Fig. 5e,f and Extended Data Fig. 3e). It is common for AbTACs to show this level of maximal degradation (*D*_max_) as it is a composite of synthesis and degradation rates^58,59,61^.

Finally, we pretreated MDA-MB-231 cells with 1 µM VH27.2 AbTAC, VH27.2–Fc and isotype controls, or PBS for 24 h. The solution containing the AbTAC or control molecules was removed, and cells were washed and then incubated with pNP-TMP substrate for 5 h. Cells primed with VH27.2 AbTAC exhibited ~36% less pNP-TMP hydrolysis relative to the PBS condition, but cells pretreated with VH27.2–Fc and isotype controls were not statistically different from the PBS condition (Fig. 5g). These results validate that degradation of ENPP1 can effectively inhibit its hydrolytic activity.

### Cryo-EM structure elucidated the allosteric binding pose

**Fig. 6:**
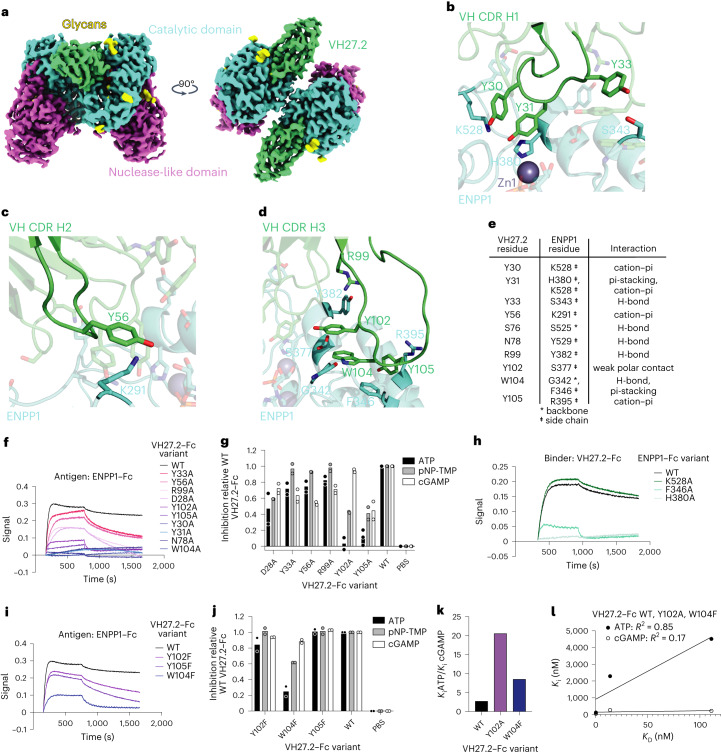
Cryo-EM reveals VH domain binding ENPP1 proximal to the catalytic site. **a**, Cryo-EM 3D reconstruction of VH27.2 bound to ENPP1 ectodomain. **b**, View of the CDR H1 epitope. **c**, View of the CDR H2 epitope. **d**, View of the CDR H3 epitope. **e**, Table summarizing interactions between VH and ENPP1 residues. **f**, Biolayer interferometry comparing binding kinetics of VH27.2–Fc alanine variants. Traces are representative of *n* = 2 independent experiments. **g**, Inhibitory potencies of VH27.2–Fc alanine variants relative to WT VH27.2–Fc treated at 500 nM for ATP, pNP-TMP and cGAMP substrates. Bar graph reports the mean for *n* = 2 independent replicates. **h**, Biolayer interferometry comparing affinity of VH27.2–Fc to WT, K528A, F346A and H380A ENPP1–Fc. Traces are representative of *n* = 2 independent experiments. **i**, Biolayer interferometry comparing binding kinetics of VH27.2–Fc phenylalanine variants. Traces are representative of *n* = 2 independent experiments. **j**, Inhibitory potencies of VH27.2–Fc phenylalanine variants relative to WT VH27.2–Fc treated at 500 nM for ATP, pNP-TMP and cGAMP substrates. Bar graph reports mean for *n* = 2 independent replicates. **k**, ATP/cGAMP *K*_i_ fold-change value for WT, Y102A and W104F VH27.2–Fc. **l**, Linear regression and *R*^2^ values correlating *K*_D_ and *K*_i_ values for ATP and cGAMP substrates for WT, Y102A and W104F VH27.2–Fc variants. Source data

**Extended Data Fig. 4:**
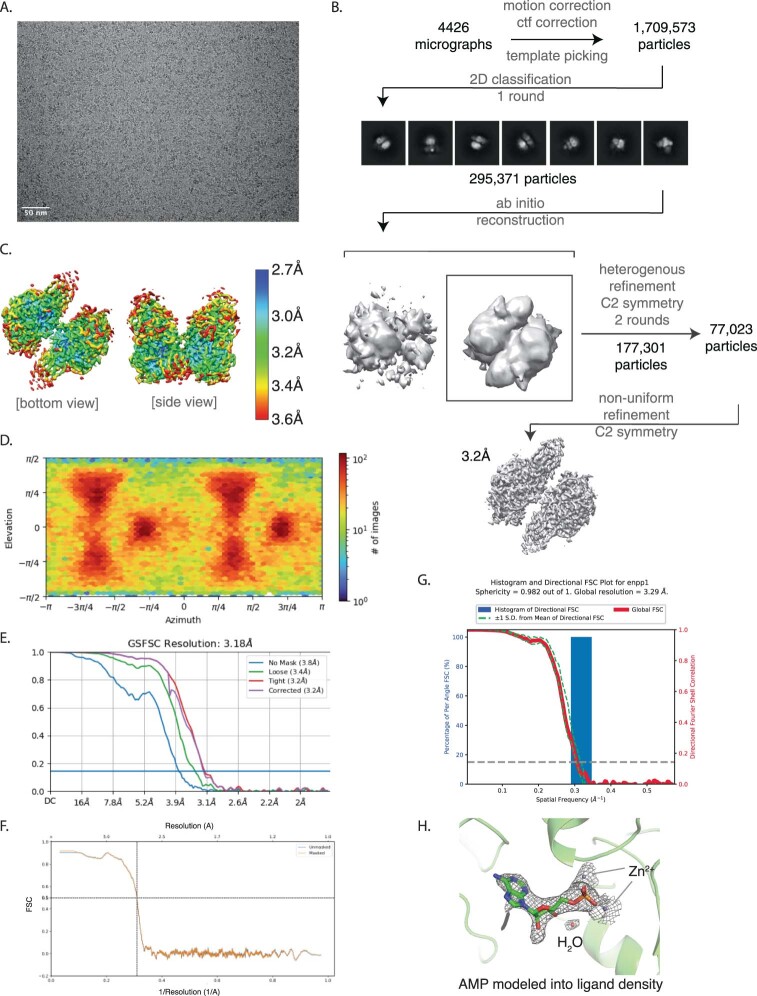
Additional data for cryo-EM data collection and processing. **a**. Representative unprocessed micrograph. **b**. Single-particle cryo-EM image processing workflow. **c**. ENPP1-VH complex colored by local resolution. **d**. Orientation angle distribution of particles in the final reconstruction. **e**. Gold-standard FSC of the final non-uniform refinement. (resolution = 3.2 Å at gold-standard FSC = 0.143). **f**. FSC curves between the experimental map and model. **g**. Histogram and Directional FSC Plot from 3DFSC. **h**. Experimental density for ligand in ENPP1 active site (mesh) with AMP modeled in (green sticks) and two zinc atoms (gray spheres).

**Extended Data Fig. 5:**
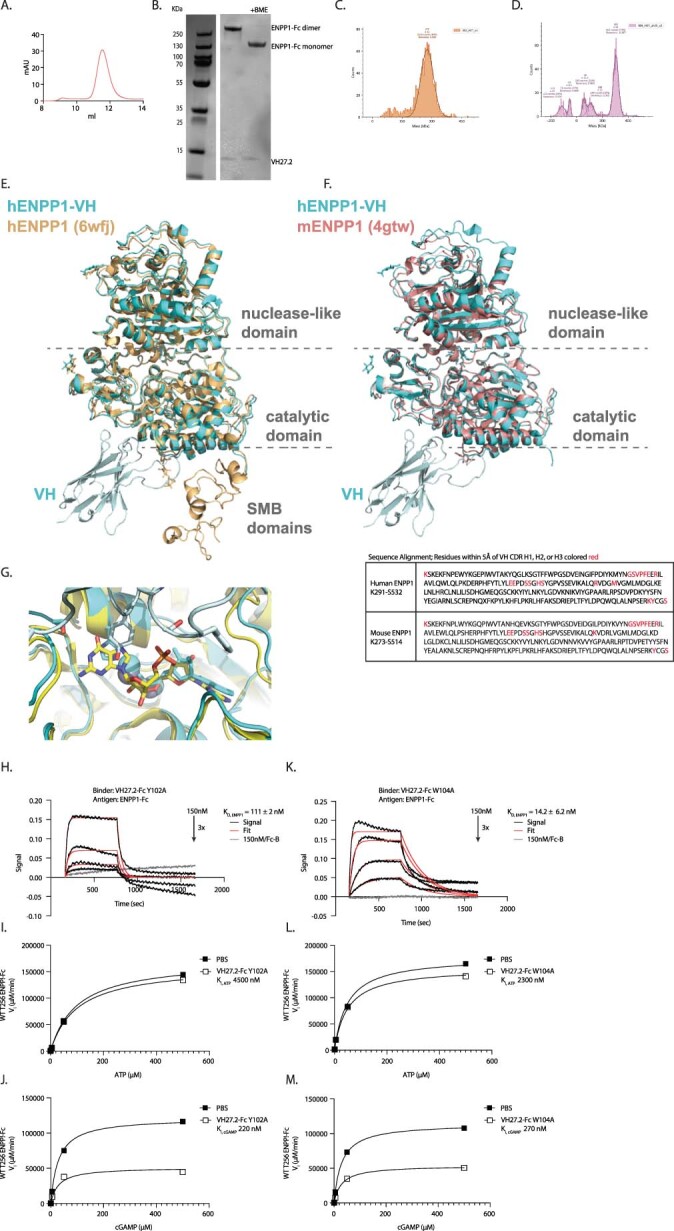
Additional data for structural and mechanistic analyses. **a–d**. Validation of VH-ENPP1 complex. Complex was purified by SEC (**a**) and co-elution of VH and ENPP1-Fc was analyzed by SDS-PAGE gel (representative of *n* = 3 independent experiments) (**b**). Mass photometry of antigen alone (**c**) and SEC-eluted complex peak (**d**) demonstrated mass shift of approximately 30 KDa suggesting 2:1 VH:antigen stoichiometry. **e**. Cryo-EM structure of VH-ENPP1 complex aligned to PDB 6wjf. **f**. Cryo-EM structure of VH-ENPP1 complex aligned to PDB 4gtw with sequence alignment of human and mouse ENPP1. Residues colored red were within 5Å of VH CDR H1, H2, or H3. **g**. Close-up of clashing with pApG when VH-ENPP1 complex (cyan) is aligned to PDB 6aek (yellow). **h**. Representative biolayer interferometry signals and fits for VH27.2-Fc Y102A (*n* = 2 independent replicates). **i, j**. Michaelis-Menten kinetic analysis for VH27.2-Fc Y102A for ATP (**i**) and cGAMP (**j**) (mean for *n* = 2 independent replicates). **k**. Representative biolayer interferometry signals and fits for VH27.2-Fc W104A (*n* = 2 independent replicates). **l, m**. Michaelis-Menten kinetic analysis for VH27.2-Fc W104A for ATP (**l**) and cGAMP (**m**) (mean for *n* = 2 independent replicates). Source data

To reveal the epitope and mechanism of allosteric inhibition, we solved a 3.2 Å global resolution cryo-EM structure of VH27.2 in complex with T256A ENPP1–Fc (Fig. 6a and Extended Data Figs. 4a–h and 5a,b; Protein Data Bank (PDB) codes: 8HGR and EMD-40047). The density for the Fc domains was not observed due to the flexible hinge and linker. The cryo-EM structure showed the expected 2:1 VH:ENPP1 homodimer stoichiometry, with a VH domain bound to each ENPP1 ectodomain configured in a head-to-head dimer (Fig. 6a and Extended Data Fig. 5c,d). The VH–ENPP1 structure was aligned to the crystal structure of full-length ENPP1 ectodomain including SMB domains (PDB code: 6wfj), and the global RMSD was 0.972 Å (Extended Data Fig. 5e)^42^. Overlaying these structures, it was apparent that the VH27.2 CDR H3 bound close to the SMB domains but without clashing. Notably, there would be insufficient space to accommodate a light chain indicating our original motivation to use small VH domains to target cryptic epitopes was imperative. The VH–ENPP1 structure was also aligned to the mENPP1 crystal structure (PDB code: 4gtw); the hENPP1 and mENPP1 catalytic domains share close to 90% homology^40,42^. The key VH-ENPP1 interface interactions were preserved, explaining species cross-reactivity (Extended Data Fig. 5f).

All three VH CDRs engaged ENPP1 with impressive shape-complementarity, binding a region close to the active site, but not occluding the site or interacting with the residues in the nucleotide-binding pocket (Fig. 6b–d)^40,41^. Density resembling a nucleotide monophosphate, which we modeled as AMP, was observed in the active site (likely left from purification because no ligand was supplied exogenously), demonstrating that VH27.2 and a nucleotide such as AMP can bind simultaneously. Substrates like ATP and cGAMP bind in the same nucleotide pocket as AMP, the product of catalysis. This suggests that these substrates would also be able to bind to the ENPP1–VH complex, consistent with the observed noncompetitive inhibition. The VH domain was skewed toward the guanosine-adjacent site, and overlaying the VH–ENPP1 structure with the crystal structure of mENPP1 complexed with 3′-5′-linked pApG (a cGAMP hydrolysis linear intermediate; PDB code: 6aek) exhibited that the VH domain would clash with the guanosine base (Extended Data Fig. 5g)^41^. However, a previous report found that the guanosine base has a degree of mobility within the pocket, such that mutations to these guanosine-adjacent resides did not completely disrupt cGAMP binding and hydrolysis^3^. Based on this positional flexibility, the VH domain overlap with these residues would not necessarily prevent cGAMP binding, as is supported by our Michaelis–Menten characterizations.

We performed epitope analysis by alanine scanning residues that formed specific interactions between VH27.2 and ENPP1 (Fig. 6e–g). These structure–function characterizations identified the following two hot spots on VH27.2 that contribute to the binding and allosteric inhibition of ENPP1: (1) CDR H1 Y30/Y31 and (2) CDR H3 Y102/W104/Y105.

The VH27.2 CDR H1 loop is internally structured through pi-stacking of Y31 and Y30, which are both within the range of cation–pi interactions with ENPP1 K528. Furthermore, VH27.2 Y31 pi-stacks with ENPP1 H380 (Fig. 6b,e). VH27.2–Fc binding to ENPP1 was abolished when Y31 or Y30 were mutated to alanine (Fig. 6f). Wild type (WT) VH27.2–Fc did not bind to ENPP1 H380A, however, retained an affinity for ENPP1 K528A, indicating that H380 is the essential contact and its aromatic interaction with VH Y31 is energetically indispensable (Fig. 6h). Together, ENPP1 H380, VH27.2 Y31, VH27.2 Y30 and ENPP1 K528 connect an extensive aromatic-cation network that greatly contributes to VH affinity.

The pi-stacking between VH27.2 Y31 and ENPP1 H380, a residue in the catalytic core of ENPP1 that coordinates Zn^2+^, may contribute to the allosteric inhibition and threefold selectivity for cGAMP inhibition over ATP (Fig. 6b,e). Because the hydrolytic activity of ENPP1 is highly dependent on the Zn^2+^ coordination of substrates, we hypothesize that VH27.2 Y31 pi-stacking with H380 could exert an inhibitory effect. Although lack of binding between VH27.2–Fc Y31A and ENPP1 prevented enzymatic inhibition assays, we note that mENPP1 H362 corresponding to hENPP1 H380 was previously discovered to be essential for cGAMP but not ATP degradation, and mENPP1 H362A was unable to coordinate Zn^2+^ for productive cGAMP hydrolysis^3^. There was Zn^2+^ density in the VH–ENPP1 complex; therefore, the pi-stacking network involving H380 may lower the pKa of the histidine residue without completely diminishing its affinity for Zn^2+^, as was observed for the more extreme histidine-to-alanine mutation^62^. Another possibility is that VH27.2 engagement at this site may perturb reaction geometry or conformational trajectory during catalysis.

Structure-guided mutagenesis uncovered a second hot spot, CDR H3, that is critical to binding affinity and inhibition of ATP hydrolysis specifically. VH27.2 CDR H3 residues Y102, W104 and Y105 pi-stack to form a hydrophobic pocket that makes several contacts with ENPP1 residues—weak polar interaction between VH27.2 Y102 and ENPP1 S377 sidechain; hydrogen bond between VH27.2 W104 and ENPP1 G342 backbone; pi-stacking between VH27.2 W104 and ENPP1 F346 and cation–pi interaction between VH27.2 Y105 and ENPP1 R395, requiring the R395 sidechain to break its salt bridge with ENPP1 E347 and rotate compared to its register in non-VH-bound ENPP1 (PDB code: 6wjf) to prevent clashing with Y105 (Fig. 6d,e). The major energetic contributions to the binding of these three aromatic residues were validated by alanine scanning. VH27.2–Fc W104A did not bind to ENPP1, and VH27.2–Fc Y102A and Y105A had weakened affinity toward ENPP1 by two orders of magnitude (Fig. 6f and Extended Data Fig. 5h).

Remarkably, 500 nM VH27.2–Fc Y102A failed to inhibit ATP hydrolysis by ENPP1 but still inhibited cGAMP and pNP-TMP hydrolysis (Fig. 6g). Comparing VH27.2–Fc Y102A to WT, the Michaelis–Menten *K*_i_ for cGAMP increased approximately fivefold to 220 nM (CI_95_: 170–290 nM). However, the *K*_i_ for ATP was greater than 4.5 µM (CI_95_: 2.8–11 µM), indicating the Y102A variant conferred extraordinary substrate selectivity (Extended Data Fig. 5i,j). This ATP-specific effect for the VH27.2–Fc Y102A variant could implicate the weak polar interaction with ENPP1 S377, or alternatively that the tyrosine residue supports the structural integrity of the hydrophobic pocket with VH27.2 W104 and Y105. To decouple these effects, we generated a phenylalanine variant for Y102 that rescued affinity and inhibition of ATP hydrolysis (Fig. 6i,j). These results indicated that the polar contact is dispensable but the hydrophobic interactions are critical for CDR conformation. Similarly, VH27.2–Fc Y105F and W104F variants restored affinity compared to the respective alanine variants, confirming the importance of this hydrophobic filling at the ENPP1 interface (Fig. 6i).

Intriguingly, the VH27.2–Fc W104F variant recapitulated the substrate bias of the Y102A variant, where VH27.2–Fc W104F was much less potent toward inhibition of ATP hydrolysis compared to cGAMP or pNP-TMP hydrolysis (Fig. 6j). The *K*_i_ value for cGAMP was 270 nM (CI_95_: 240–300 nM), but the *K*_i_ value for ATP was 2.3 µM (CI_95_: 1.2–10 µM; Extended Data Fig. 5l,m). While WT VH27.2–Fc was 2.7-fold selective for inhibiting cGAMP hydrolysis over ATP hydrolysis, VH–Fc Y102A and W104F were ~20.6- and ~8.5-fold selective, respectively (Fig. 6k). Although WT, Y102A and W104F VH27.2–Fc had dramatically different binding affinities (*K*_D_ = 0.7, 111 and 14.2 nM, respectively), there was no correlation (*R*^2^ = 0.17) between affinity and the *K*_i_ value for cGAMP. In contrast, there was a strong correlation (*R*^2^ = 0.85) when comparing affinity and the *K*_i_ value for ATP, indicating that destabilization of CDR H3 disproportionately detuned the potency for ATP (Fig. 6l and Extended Data Fig. 5h–m). Mechanistically, the W104F variant disrupts the hydrogen bond to ENPP1 G342 backbone carbonyl. Phenylalanine is also unable to fill the hydrophobic hole from the tryptophan and might weaken the aromatic interaction with ENPP1 F346. WT VH27.2–Fc had reduced affinity for ENPP1 F346A variant, validating that the pi-stacking between ENPP1 F346 and VH27.2 W104 is an important energetic component (Fig. 6h).

Comprehensively, these mutagenesis results suggested that VH27.2 W104 is imperative for the ATP-inhibitory mechanism, and the hydrophobic filling of Y102 supports the correct conformation of W104. Furthermore, VH27.2–Fc Y102A and W104F variants minimally affected the *K*_i_ for cGAMP, implying that CDR H1 engagement with ENPP1 H380 has a dominant role in the inhibitory mechanism for cGAMP. Therefore, structure–activity analysis revealed that the VH CDRs engage distinct sites on ENPP1 that separately modulate the hydrolysis of cGAMP or ATP. These discoveries may inspire designs of new small molecule and biologic inhibitors that interrogate the same allosteric epitopes as our VH domain.

## Discussion

As potent small-molecule inhibitors for ENPP1 begin to show exciting translational results, biologics provide a complementary mode of inhibition with different strengths^2^. First, drug localization to tumor cells is important for minimizing potential off-tumor toxicities and mitigating drug sequestration by soluble ENPP1. Small molecules typically distribute systemically, and it can be challenging to engineer them for greater tumor specificity. In contrast, we recombinantly expressed the VH inhibitor as a bispecific molecule dual-targeting high PD-L1 and ENPP1 expression, substantially improving the cellular IC_50_ value without changing the IC_50_ value in plasma. Bispecific molecules cotargeting other tumor biomarkers can be engineered to address various cancer types.

Second, studies have indicated that combining ENPP1 inhibitors with checkpoint inhibitors increases their therapeutic index against immune ‘cold’ tumors^11,13,14^. It is difficult to build drug-like large bifunctional small molecules. In contrast, straightforward protein engineering approaches allowed the construction of a bispecific with VH27.2 and Envafolimab. ENPP1 is also an antimetastatic target and could be used in bifunctional formats with other therapeutics^4–6,8,9^. For example, OS becomes fatal when the cancer progresses to metastasis^63^. As HER2 and ENPP1 are overexpressed in OS, a bispecific molecule combining the ENPP1 inhibitor with a second clinical antibody such as trastuzumab could be an opportunity to treat this aggressive cancer.

Third, antibody-based ENPP1 inhibitors also function as antigen-directed immunotherapies. NK cells and macrophages will target tumor cells bound with the Fc effector active forms for ADCC, and the ENPP1 inhibitor in BiTE format robustly activated T cells. Additionally, VH domains are useful because of their stable expression as chimeric antigen receptor-T cells (CAR–Ts). We hypothesize that layering cGAMP activation with antigen-directed killing could potentiate the therapeutic efficacy of biologic inhibitors compared to small molecules that have only a single mechanism of action. As the VH inhibitor is cross-reactive with mENPP1, these immunotherapeutic synergies will be evaluated using immunocompetent mice in future studies. One important consideration is that ENPP1 is also expressed in some normal tissues; therefore, potential off-tumor toxicities of anti-ENPP1 immunotherapies will require investigation. Bispecific CAR or BiTE modalities that cotarget a second tumor antigen could enhance localization to tumor cells over healthy cells.

Inhibition via targeted degradation is another therapeutic approach enabled by the panel of anti-ENPP1 VH domains. The VH27.2 AbTAC still retains its function as an inhibitor, so we believe that degradation will be additive. We have focused on inhibiting ENPP1 biochemical activity; however, ENPP1 is known to negatively regulate insulin signaling through direct binding to the insulin receptor^36,37,40^. It is possible that degrading ENPP1 to eliminate this interaction could have therapeutic relevance to insulin resistance and type 2 diabetes.

Finally, the modularity of the VH binders for substrate-selective inhibition is a key advantage over current small-molecule ENPP1 inhibitors. Using structure-guided mutagenesis, we determined that CDR H3 has a substantial role in blocking ATP but not cGAMP hydrolysis, and we suspect that the CDR H1 interaction with H380 is substantial to the mechanism of inhibition for cGAMP degradation, as would be consistent with previous reports^3^. We demonstrated that the VH CDRs could be modulated to generate cGAMP-selective inhibitors, and with further affinity maturation and optimization, these will be valuable reagents for studying cGAMP signaling in disease and potential therapeutics with fewer liabilities. Additionally, the discovery of these separate allosteric pockets that decouple ATP and cGAMP hydrolytic mechanisms can aid future designs of small molecule and biologic inhibitors with greater potencies and specialized activities. In summary, our studies exemplify that unbiased screening for protein binders in different size formats coupled with detailed biochemical and structural characterization can reveal new mechanistic insights and molecular opportunities for biologic and small molecule discovery.

## Methods

### Cell culture

PDX-derived OS384 cells were engineered with a plasmid containing SG RNA or ENPP1 guide RNA (KO). Cells were cultured in DMEM containing 10% FBS, 1× glutamine and 1% penicillin/streptomycin (pen/strep) at 37 °C and 5% CO_2_. Jurkat cells expressing NFAT–GFP reporter were cultured in RPMI containing 10% FBS, 1% pen/strep and 2 mg ml^−1^ Geneticin at 37 °C and 5% CO_2_. MDA-MB-231 cells were cultured in DMEM containing 10% FBS and 1% pen/strep at 37 °C and 5% CO_2_. The OS384 PDX-derived and MDA-MB-231 cell lines were validated by short tandem repeat (STR) testing, and NFAT–GFP Jurkat cell line was purchased from Thermo Fisher Scientific.

### Mammalian expression of proteins (ENPP1–Fc, VH–Fc, BiTE and AbTAC)

HEK-293 EXPI (Expi293) cells were cultured in Expi293 media (Gibco) at 37 °C and 8% humidity with orbital shaking at 250 r.p.m. ENPP1–Fc or VH–Fc were cloned into a pFUSE vector (InvivoGen) with upstream IL-2 secretion signal. Cells were transfected at 3 M ml^−1^ density using ExpiFectamine Transfection kit (Gibco), according to manufacturer protocols. To biotinylate the AviTag of ENPP1–Fc, Expi293 cells expressing BirA were used and 0.05 mM biotin was included in the media at the time of transfection. Seventy-two to 120 h after the addition of enhancers, the supernatant was collected (30 min, 4,000*g*) and filtered using 0.45-micron filters. Proteins were purified with Protein A affinity chromatography or nickel resin and buffer exchanged into PBS using appropriate MW spin filters (Amicon). Protein purity was assessed using SDS–PAGE, and proteins were stored at −80 °C.

### Bacterial expression of proteins (single-domain VH)

VH were expressed in *Escherichia coli* C43(DE3) Pro+ pTUM+ using optimized autoinduction media. Bacterial pellets were lysed with B-PER (Thermo Fisher Scientific) containing protease inhibitors (MilliporeSigma), and lysates were purified using Protein A affinity chromatography and buffer exchanged into PBS using 3 MW or 10 MW spin filters (Amicon). Protein purity was assessed using SDS–PAGE, and proteins were stored at −80 °C.

### Phage selection with VH-phage library

Phage display using a VH-phagemid library was performed according to previously established protocols^44^.

For the standard selection, biotinylated antigen or biotinylated Fc was immobilized to streptavidin-coated magnetic beads (Promega). For each round, the phage library was first cleared by incubating with Fc to remove nonspecific binders, and subsequently, the positive selection was performed using the antigen. Phage-binding antigens were washed three times and eluted by treatment with 2 µg ml^−1^ TEV protease. XL-1 blue *E. coli* were infected with eluted phage and allowed to propagate overnight. In total, four rounds of selection were performed with increasing antigen and clear stringency. All steps were carried out in Tris-buffered saline (TBS) buffer containing 0.02% TWEEN-20, 0.2% BSA, 200 µM zinc chloride, 2 mM calcium chloride and 75 mM sodium chloride. After four rounds, single XL-1 blue colonies were picked and evaluated by ELISA and Sanger sequencing.

For the substrate competitive selection, the procedure above was performed, but instead of TEV treatment, the bound phage were eluted by incubating with 10 mM ATP for 30 min at room temperature.

### VH maturation via AHEAD

VH27 was integrated into the AHEAD yeast strain (yAW301), as previously reported^47^. VH27 was first subcloned into the pAW240 integration plasmid, linearized via restriction digest and transformed into chemically competent yAW301 using Frozen-EZ Yeast Transformation II kit (Zymo). After 3 d of growth on selective Synthetic Complete without histidine, leucine, uracil, tryptophan, methionine and cysteine and supplemented with 2% glucose (SC-HLUWMC) dropout media (US Biologics), single colonies were grown to saturation in SC-HLUWMC. Incorporation of VH27 was validated by Sanger sequencing. VH27 was autonomously diversified by passaging transformed yAW301 through 2–3 1,000× fold dilutions in SC-HLUW with 2% glucose. For FACS experiments, VH27 expression was induced in yeast by switching to SC-HLUW with 2% galactose for 48 h. Yeast was labeled with biotinylated ENPP1–Fc for 1–2 h, with round 1 starting at 65 nM and every subsequent round at half the previous concentration. To prevent ligand depletion and allow binding reactions to reach equilibrium, yeast cell counts, reaction volumes and incubation times were further adjusted. After washing, yeast were stained with 1,000× dilution of anti-HA-AF488 (Thermo Fisher Scientific) and streptavidin-647 (Thermo Fisher Scientific), washed again and subjected to FACS using an Aria II instrument. During each round, ~2 × 10^7^ cells were stained and used to sort out 200–2,000 cells. To avoid the selection of variants with mutations in VH27’s HA-tag (fused to VH27 to measure display level by anti-HA-AF488), a strict floor on HA signal was set such that only the top ~15% of clones on the HA signal axis were sorted for ENPP1–Fc labeling. Omitting HA-tag variants was important because they falsely appear as improved ENPP1 binders because they disable the HA-tag display level signal to which the ENPP1–Fc signal is normalized. Cells were sorted into 3 ml of SC-HLUW with 2% glucose, grown to saturation (~3 d) and then subjected to the next round.

### BLI

BLI data were collected using an OctetRED384 (ForteBio) instrument. To analyze the association and dissociation kinetics of purified binders, streptavidin biosensor tips (Sartorius) were loaded with antigen, blocked with 5 µM biotin, dipped into protein analyte in solution for 600 s (association) and finally dipped into buffer for 900 s (dissociation). For epitope binning experiments, a biosensor loaded with ENPP1–Fc was blocked with 5 µM biotin, dipped into a solution containing 25 nM binder 1 until saturation (600 s) and then immediately dipped into a solution containing 25 nM binder 1 and 25 nM binder 2 (600 s). Experiments for ENPP1 antigen were performed using TBS buffer containing 0.02% TWEEN-20, 0.2% BSA, 200 µM zinc chloride, 2 mM calcium chloride and 75 mM sodium chloride. All other antigens were assayed using PBS containing 0.02% TWEEN-20 and 0.2% BSA. Data were collected using ForteBio Octet Data Acquisition software (v12.01.11) and analyzed using ForteBio Octet analysis software (v12.0), and kinetic parameters were determined based on a 1:1 monovalent binding model.

### Flow cytometry

Cells were lifted with versene, washed in PBS and resuspended in PBS with 3% BSA. Cells were stained with binder at indicated concentrations in PBS with 3% BSA for 1 h at 4 °C. Cells were washed twice with PBS with 3% BSA. Cells were then incubated with ProtA-647 secondary (Thermo Fisher Scientific) at 1:1,000 dilution in PBS with 3% BSA for 30 min at 4 °C. Cells were washed three times in PBS with 3% BSA cells and resuspended in PBS for flow cytometry analysis using a Beckman Coulter Cytoflex Flow Cytometer and CytExpert (v2.3.1.22) software. Data were processed with FlowJo (v10.8.1) software, and EC_50_ curves were calculated using GraphPad PRISM 9.0 ‘Sigmoidal, 4PL’ nonlinear fit with Hill coefficient set to greater than 1.

### Recombinant hENPP1- and mENPP1-inhibition assays

T256 ENPP1–Fc was cloned and used for recombinant enzyme activity assays. The Michaelis–Menten reaction conditions used were 5 nM ENPP1–Fc, 300 nM inhibitor (or PBS) and a titration of 500, 50, 5 or 0.5 µM ATP (Thermo Fisher Scientific) or cGAMP (InvivoGen). The reaction buffer was TBS containing 0.02% TWEEN-20, 0.2% BSA, 200 µM zinc chloride, 2 mM calcium chloride and 75 mM sodium chloride. Every 2 min for a total of 8 min, the reactions were quenched by heating to 95 °C for 10 min. Cell-Titer Glo (Promega) or AMP-Glo (Promega) was performed according to manufacturer protocol to determine the amount of ATP remaining or AMP produced from cGAMP hydrolysis. The initial velocity was determined by averaging the velocities calculated over 4–8 min (ATP) and 2–8 min (cGAMP) at each substrate concentration. The Michaelis–Menten *K*_i_ was analyzed using GraphPad PRISM 9.0 ‘enzyme kinetics-noncompetitive inhibition’ model.

To assess single-point inhibition values for alanine and phenylalanine variants, 5 nM ENPP1–Fc (T256 or catalytically dead control), 500 nM inhibitor (or PBS) and substrate (ATP = 2.5 mM, cGAMP = 1 mM and pNP-TMP = 1 mM) were incubated in reaction buffer at 25 °C for 30 min, quenched by heating to 95 °C for 10 min and assayed by Cell-Titer Glo (ATP), AMP-Glo (cGAMP) or absorbance at 405 nm (pNP-TMP).

mENPP1 was purified as previously described^2,40^. In total, 10 nM mENPP1 and 500 nM VH27/T75I/A98I-Fc/Fc isotype or PBS were incubated with 2.5 mM ATP or 1 mM cGAMP in reaction buffer at 25 °C for 30 min. Reactions were quenched by heating at 95 °C for 10 min, and ENPP1 activity was measured by Cell-Titer Glo (ATP) or AMP-Glo (cGAMP).

### Human- and mouse-recovered plasma IC_50_ assay

Recovered human plasma (sodium heparin anticoagulant) from three donors was purchased from ZenBio (Donor 1:031620A, Donor 2:PL070720AC and Donor 3:PL070720X). Plasma from three male C57BL/6J mice was prepared with sodium heparin anticoagulant, as previously described^2^. Final reactions were 10 µl and contained 65% plasma, 1 mM cGAMP and inhibitor or control molecule at the indicated concentration. A reaction without cGAMP was used as a control to subtract the baseline AMP signal. Reactions were incubated for 90 min (or 24 h when specified). Samples were diluted tenfold in PBS and either measured immediately or quenched and stored for later analysis by centrifuging in a 10 MW spin filter. AMP produced through cGAMP hydrolysis was measured using AMP-Glo assay. Values were corrected by subtracting the background signal of plasma without supplemented cGAMP, and data were normalized to the PBS condition. IC_50_ value for each donor was analyzed using GraphPad PRISM 9.0 ‘Dose-response-Inhibition (four parameters)’.

### Differential scanning fluorimetry

Purified VH protein was diluted to 1 µM in PBS buffer containing 4× Sypro Orange (Invitrogen). BME was supplemented to 6.25% final dilution for samples assayed in reducing conditions. Samples were heated from 30 °C to 95 °C with 0.3 C/30 s ramp rate, and fluorescent emissions at 490 nm and 575 nm were continuously recorded. Roche LC480 LightCycler and associated Thermal Shift Analysis software (v2.0.2015.813) were used for data collection and *T*_m_ calculations.

### SEC

SEC was performed using an Agilent HPLC 1260 Infinity II LC System and either AdvanceBio column (300 Å, 2.7 µM; Agilent) or TSKgel SuperSW mAb HTP column (4 µM; Tosoh Bioscience). Fluorescence was detected using excitation 285 nm and emission 340 nm.

### Extracellular cGAMP IC_50_ ELISA

MDA-MB-231 cells were plated at 2 K cell/well 24 h before cell treatments. Assay was performed in serum-free DMEM/F12 media that contains zinc sulfate. Cells were washed with media and resuspended in media containing the inhibitor (or Fc isotype or PBS) and allowed to incubate for 5 min before cGAMP was added at a final concentration of 50 µM. cGAMP was also added to a well containing only media without cells as a maximum concentration control. Cells were placed at 37 °C and 5% CO_2_ for 12 h. To collect, 2 µl of media was diluted 50-fold in PBS and centrifuged in a 10 MW spin filter. To assay cGAMP remaining in the media by cGAMP ELISA (Cayman Chemical), the sample was diluted another 75× using Immunoassay Buffer-C (Cayman Chemical), and the ELISA was performed according to the manufacturer protocol. The level of cGAMP remaining in each sample was normalized to the range between the PBS control (full enzymatic activity) and cGAMP in media without cells (no enzymatic activity). Data were analyzed using GraphPad PRISM 9.0 ‘Dose-response-Inhibition (four parameters)’. For the biparatopic molecule, the 3 µM concentration was excluded from the IC_50_ calculation.

### Extracellular pNP-TMP IC_50_ assay

MDA-MB-231 cells were plated at 2 K cell/well 36 h before cell treatments. Assay was performed in serum-free and phenol-red-free DMEM/F12 media. Cells were washed with media and resuspended in media containing the inhibitor (or Fc isotype or PBS) and incubated for 10 min. pNP-TMP substrate (Sigma Aldrich) was added at a final concentration of 200 µM. Cells were placed at 37 °C and 5% CO_2_ for 5 h, and absorbance at 405 nm was measured. Data were normalized using the PBS treatment. Data were analyzed using GraphPad PRISM 9.0 ‘Dose-response-Inhibition (four parameters)’. For the biparatopic molecule, the 3 µM concentration was excluded from the IC_50_ calculation.

### NFAT–GFP Jurkat assay

Streptavadin-coated magnetic beads (Promega) were incubated with 0, 10 or 100 nM ENPP1–Fc antigen for 30 min at room temperature followed by three washes. In each assay well, 50 K Jurkat NFAT–GFP cells were plated in serum-free RPMI and treated with 20 µl of beads and 10 nM BiTE. Conditions with no beads and without BiTE (PBS) were included for controls. After incubating for 20 h at 37 °C and 5% CO_2_, magnetic beads were removed and cellular GFP expression was analyzed by flow cytometry.

### AbTAC

MDA-MB-231 cells were plated 8 K/well in 48-well culture plates and incubated at 37 °C for 24 h. Cells were treated with the indicated concentration of AbTAC or control molecule in serum-free DMEM/F12 media and incubated at 37 °C and 5% CO_2_ for 24 h. Cells were washed with PBS, and lysates were collected with RIPA (Sigma Aldrich) containing protease inhibitor. Samples for gel electrophoresis were prepared by boiling lysate in NuPAGE loading dye containing BME. Proteins were transferred from the gel to a polyvinylidene difluoride (PVDF) membrane using iBlot reagents and instruments (Thermo Fisher Scientific). Membranes were blocked and subsequently stained with anti-ENPP1 antibody (Abcam, 223268) and anti-actin antibody (Santa Cruz Biotechnology, sc-47778) at 1:1,000 dilutions at 4 °C overnight. Membranes were then stained with LICOR secondary antibodies (goat anti-rabbit 680 and goat anti-mouse 800) at 1:5,000 dilutions for 1 h at room temperature and imaged using the LICOR Odyssey instrument with Image Lab (v 5.0) and Image Studio software (v 5.2). Data were analyzed using Image Studio Lite software.

To measure the effect of ENPP1 degradation on extracellular pNP-TMP hydrolysis, 4 K MDA-MB-231 cells were plated in culture media in 96-well plates and incubated at 37 °C and 5% CO_2_ for 24 h. Cells were treated with 1 µM VH27.2 AbTAC or control molecule prepared in serum-free and phenol-red-free DMEM/F12 media and incubated at 37 °C and 5% CO_2_ for 24 h. The solution containing the AbTAC or control molecules was removed, cells were washed twice with fresh serum-free and phenol-red-free DMEM/F12 media and serum-free and phenol-red-free DMEM/F12 media containing 200 µM pNP-TMP substrate was added. Cells were placed at 37 °C and 5% CO_2_ for 5 h, and absorbance at 405 nm was measured. Data were normalized to the PBS treatment.

### Structural sample preparation, data collection and processing

T256A ENPP1–Fc and 10× VH27/T75I/A89V were incubated at 4 °C for 1 h. The complex was injected onto an Äkta Pure system (GE HealthCare), and the peak was isolated by SEC using a Superdex 200 Increase 10/300 GL column (Extended Data Fig. 5a). Presence of both VH and antigen in the eluted peak was confirmed by SDS–PAGE gel (Extended Data Fig. 5b). Stoichiometry of the complex was characterized by Refeyn mass photometry (Extended Data Fig. 5c,d).

For cryo-EM grids preparation, 2 mg ml^−1^ T256A ENPP1–Fc was mixed with 10× VH27/T75I/A89V, and the mixture was directly applied to glow-discharged 300 mesh gold grids (Quantifoil R1.2/1.3) and vitrified using an FEI Vitrobot Mark IV (Thermo Fisher Scientific). Data were collected on a Titan Krios (SLAC/Stanford) operated at 300 keV using a Gatan K3 direct electron detector in counting mode, with 0.8677 Å pixel size. A total of 4,426 movies were obtained. Each stack movie was recorded for a total of 2.5 s with 0.05 s per frame. The dose rate was 1.14 electrons/Å^2^/subframe, resulting in an accumulated dose of 57 electrons per Å^2^. The data were collected using SerialEM^64^.

Data processing was done in cryoSPARC2 v3.3.2. Dose-fractionated movies (Extended Data Fig. 4a) were subjected to beam-induced Patch Motion correction followed by Patch-Based Contrast Transfer Function (CTF) parameters estimation. After that, 3,414 movies with CTF fit better than 4 Å were selected. Particle autopicking with Blob Picker, 2D classification and 3D refinements were performed in cryoSPARC. The autopicked particles were first subjected to 2D classification; 2D classes that look like protein complexes were selected for ab initio reconstruction and heterogenous refinement (Extended Data Fig. 4b–d). The best 3D class (highest estimated resolution and good orientation distribution) was selected, and the particles are subjected to nonuniform refinement. Resolution of the final map for the ENPP1–VH complex was 3.2 Å (masked), as determined by the gold-standard Fourier shell correlation cutoff of 0.143 (Extended Data Fig. 4e).

The structure of human ENPP1 (PDB code: 6WFJ) and a homology model of VH27 (based on PDB code: 7JWB) were rigid body fit into the local resolution estimated map using University of California, San Francisco (UCSF) Chimera^65^, and then manually adjusted using Coot and real space refined in PHENIX^66,67^. Geometries of the structure models were validated using MolProbity^68^. The Fourier shell correlation curves were calculated between the refined models and full maps using PHENIX (Extended Data Fig. 4f). Structural figures were generated using PyMOL and UCSF Chimera^69^. 3DFSC analysis was done using the online server (https://3dfsc.salk.edu/; Extended Data Fig. 4g)^70^. Density was present in the active site of ENPP1 that resembled a nucleotide monophosphate, although no ligand was included in the experiment. This density was fit to AMP based on previous knowledge that ENPP1 binds adenosine more tightly than the other nucleosides (Extended Data Fig. 4h)^40^.

The parameters used for data collection and processing are presented in Supplementary Table 1.

### Reporting summary

Further information on research design is available in the Nature Portfolio Reporting Summary linked to this article.

## Online content

Any methods, additional references, Nature Portfolio reporting summaries, source data, extended data, supplementary information, acknowledgements, peer review information; details of author contributions and competing interests; and statements of data and code availability are available at 10.1038/s41589-023-01368-5.

### Supplementary information

Supplementary Information Supplementary Table 1.

Reporting Summary

### Source data

Source Data Fig. 1 Statistical source data.

Source Data Fig. 2 Statistical source data.

Source Data Fig. 3 Statistical source data.

Source Data Fig. 4 Statistical source data.

Source Data Fig. 5 Statistical source data.

Source Data Fig. 6 Statistical source data.

Source Data Extended Data Fig. 2 Statistical source data.

Source Data Extended Data Fig. 5 Statistical source data.

## Data Availability

Cryo-EM structural data are deposited in the Protein Data Bank (PDB code: 8GHR) and Electron Microscopy Data Bank (EMD-40047). Additional PDB referenced: 6wfj, 4gtw, 6aek and 7jwb. The paper contains extended data, a supplementary table and source data. Additional information is available upon request. Source data are provided with this paper.
