# Supplementary material for: Discovery of VH domains that allosterically inhibit ENPP1
Source: Nat Chem Biol. 2023 Jul 3;20(1):30–41. doi: 10.1038/s41589-023-01368-5 (PMC10746542; doi:10.1038/s41589-023-01368-5)
Supplement: Supplementary file 1 — Supplementary Table 1. [file 41589_2023_1368_MOESM1_ESM.pdf]

nature chemical biology

Article

https://doi.org/10.1038/s41589-023-01368-5

Discovery of VH domains that allosterically inhibit ENPP1

In the format provided by the authors and unedited

Contents

Supplementary Table 1

Supplementary Table 1. Parameters for cryo-EM data collection, processing, and refinement.

	VH-T256A ENPP1 complex (EMDB-40047) (PDB 8GHR)
**Data collection and processing**	
Magnification	130,000
Voltage (kV)	300
Electron exposure (e–/Å^2)	57
Defocus range (μm)	0.7-2
Pixel size (Å)	0.8677
Symmetry imposed	C2
Initial particle images (no.)	1709573
Final particle images (no.)	77023
Map resolution masked (Å)*	3.2
FSC threshold	0.143
Map resolution range (Å)	2.73-30
**Refinement**	
Initial model used (PDB code)	6WFJ
Model resolution masked (Å)	3.23
FSC threshold	0.5
Model resolution range (Å)	3.2-31.89
Map sharpening *B* factor (Å^2)	-107.54
Model composition	
Non-hydrogen atoms	13864
Protein residues	1716
Ligands	20
B factors (Å^2)	
Protein	45.44
Ligand	40.18
R.m.s. deviations	
Bond lengths (Å)	0.002
Bond angles (°)	0.576
Validation	
MolProbity score	1.53
Clashscore	5.07

Poor rotamers (%)	0
Ramachandran plot	
Favored (%)	96.14
Allowed (%)	3.86
Disallowed (%)	0

*Unmasked map resolution = 3.8 Å
