# Supplementary material for: Discovery of VH domains that allosterically inhibit ENPP1
Source: Nat Chem Biol. 2023 Jul 3;20(1):30–41. doi: 10.1038/s41589-023-01368-5 (PMC10746542; doi:10.1038/s41589-023-01368-5)
Supplement: Supplementary file 2 — Reporting Summary [file 41589_2023_1368_MOESM2_ESM.pdf]

nature portfolio

Corresponding author(s): James A. Wells

Last updated by author(s): May 18, 2023

Reporting Summary

Nature Portfolio wishes to improve the reproducibility of the work that we publish. This form provides structure for consistency and transparency in reporting. For further information on Nature Portfolio policies, see our Editorial Policies and the Editorial Policy Checklist.

Statistics

For all statistical analyses, confirm that the following items are present in the figure legend, table legend, main text, or Methods section.

n/a	Confirmed	
☐	☒	The exact sample size (n) for each experimental group/condition, given as a discrete number and unit of measurement
☐	☒	A statement on whether measurements were taken from distinct samples or whether the same sample was measured repeatedly
☐	☒	The statistical test(s) used AND whether they are one- or two-sided<br>*Only common tests should be described solely by name; describe more complex techniques in the Methods section.*
☒	☐	A description of all covariates tested
☒	☐	A description of any assumptions or corrections, such as tests of normality and adjustment for multiple comparisons
☐	☒	A full description of the statistical parameters including central tendency (e.g. means) or other basic estimates (e.g. regression coefficient) AND variation (e.g. standard deviation) or associated estimates of uncertainty (e.g. confidence intervals)
☐	☒	For null hypothesis testing, the test statistic (e.g. F, t, r) with confidence intervals, effect sizes, degrees of freedom and P value noted<br>*Give P values as exact values whenever suitable.*
☒	☐	For Bayesian analysis, information on the choice of priors and Markov chain Monte Carlo settings
☒	☐	For hierarchical and complex designs, identification of the appropriate level for tests and full reporting of outcomes
☒	☐	Estimates of effect sizes (e.g. Cohen's d, Pearson's r), indicating how they were calculated

Our web collection on statistics for biologists contains articles on many of the points above.

Software and code

Policy information about availability of computer code

Data collection	CytExpert (v 2.3.1.22) was used for flow cytometry acquisition. ForteBio Octet Data Acquisition (v 12.02.11) was used for biolayer interferometry. Image Studio Software (v 5.2) and Image Lab (v 5.0) were used for western blot scanning. Roche LC480 LightCycler (v 1.5.0) was used for DSF acquisition. SerialEM was used for cryo-EM data collection.
Data analysis	FlowJo (v 10.8.1) software was used for flow cytometry analysis. ForteBio Data Analysis (v 12.0) was used for biolayer interferometry analysis. Image Studio Lite Software was used for immunoblot analysis. Roche LC480 LightCycler Thermal Shift Analysis (v 2.0.2015.813) software was used for DSF analysis. cryoSPARC2 v3.3.2, Coot, PHENIX, MolProbity, Pymol, and Chimera were used for cryo-EM anlaysis.GraphPad Prism (v 9) software was used for data analysis.

For manuscripts utilizing custom algorithms or software that are central to the research but not yet described in published literature, software must be made available to editors and reviewers. We strongly encourage code deposition in a community repository (e.g. GitHub). See the Nature Portfolio guidelines for submitting code & software for further information.

Data

Policy information about availability of data

All manuscripts must include a data availability statement. This statement should provide the following information, where applicable:

- Accession codes, unique identifiers, or web links for publicly available datasets
- A description of any restrictions on data availability
- For clinical datasets or third party data, please ensure that the statement adheres to our policy

Cryo-EM structural data are deposited in the Protein Data Bank (PDB 8GHR) and Electron Microscopy Data Bank (EMD-40047). Additional PDB referenced: 6wfj, 4gtw, 6aek, and 7jwb. Paper contains extended data, supplementary table, and source data. Additional information is available upon request.

Human research participants

Policy information about studies involving human research participants and Sex and Gender in Research.

Reporting on sex and gender	Study did not involve human research.
Population characteristics	Study did not involve human research.
Recruitment	Study did not involve human research.
Ethics oversight	Study did not involve human research.

Note that full information on the approval of the study protocol must also be provided in the manuscript.

Field-specific reporting

Please select the one below that is the best fit for your research. If you are not sure, read the appropriate sections before making your selection.

☒ Life sciences ☐ Behavioural & social sciences ☐ Ecological, evolutionary & environmental sciences

For a reference copy of the document with all sections, see nature.com/documents/nr-reporting-summary-flat.pdf

Life sciences study design

All studies must disclose on these points even when the disclosure is negative.

Sample size	Statistical methods to predetermine sample size were not conducted. Sample size of data collected were chosen such that interpretation and statistical significance of the data were clear. The rationale was based on previous lab experience with these assays in terms of variability and detecting statistical significance.
Data exclusions	No data were excluded.
Replication	Samples were tested in at least biological replicate or triplicate as indicated. All attempts at replication were successful.
Randomization	Experimental study groups were not used therefore randomization is not relevant.
Blinding	Experimental study groups were not used therefore blinding is not relevant.

Reporting for specific materials, systems and methods

We require information from authors about some types of materials, experimental systems and methods used in many studies. Here, indicate whether each material, system or method listed is relevant to your study. If you are not sure if a list item applies to your research, read the appropriate section before selecting a response.

Materials & experimental systems

n/a	Involved in the study
☐	☒ Antibodies
☐	☒ Eukaryotic cell lines
☒	☐ Palaeontology and archaeology
☒	☐ Animals and other organisms
☒	☐ Clinical data
☒	☐ Dual use research of concern

Methods

n/a	Involved in the study
☒	☐ ChIP-seq
☐	☒ Flow cytometry
☒	☐ MRI-based neuroimaging

Antibodies

Antibodies used

VH24, VH27, VH27.2, VH38, anti-ENPP1 (Abcam 223268, 1:1000), anti-actin (Santa Cruz Biotechnologies sc-47778, 1:1000), anti-mouse (LICOR 926-32210, 1:5000), anti-rabbit (LICOR 926-68071, 1:5000)

Validation

VH24, VH27, VH27.2, VH38 were derived in-house using a human VH-phage synthetic library and validated by biolayer interferometry, flow cytometry, and biophysical stabilitiy in this study. Additional antibodies were purchased commercially.
anti-ENPP1 (Abcam 223268):https://www.abcam.com/enpp1pc1-antibody-epr22262-22-ab223268.html
anti-actin (Santa Cruz Biotechnologies):https://www.scbt.com/p/beta-actin-antibody-c4
anti-mouse (LICOR):https://www.licor.com/bio/reagents/irdye-800cw-goat-anti-mouse-igg-secondary-antibody
anti-rabbit (LICOR):https://www.licor.com/bio/reagents/irdye-680rd-goat-anti-rabbit-igg-secondary-antibody

Eukaryotic cell lines

Policy information about cell lines and Sex and Gender in Research

Cell line source(s)

OS384 CRISPR knockout cell lines were generated and authenticated in the lab of E. Alejandro Sweet-Cordero. NFAT-Jurkat cell line was purchased authenticated from Thermo. Expi293 cell line was purchased from Thermo. MDA-MB-231 cell line was purchased authenticated from UCSF Cell and Genome Engineering Core.

Authentication

OS384 and MDA-MB-231 cell lines were validated by STR testing. NFAT-Jurkat cell line was authenticated by supplier (mycoplasma and sterility). Expi293 cell line was authenticated by supplier (mycoplasma and sterility).

Mycoplasma contamination

Cell lines tested negative for mycoplasma contamination.

Commonly misidentified lines
(See ICLAC register)

None used

Flow Cytometry

Plots

Confirm that:

☒ The axis labels state the marker and fluorochrome used (e.g. CD4-FITC).

☒ The axis scales are clearly visible. Include numbers along axes only for bottom left plot of group (a 'group' is an analysis of identical markers).

☐ All plots are contour plots with outliers or pseudocolor plots.

☐ A numerical value for number of cells or percentage (with statistics) is provided.

Methodology

Sample preparation

Cells were lifted with versene, washed with PBS, and resuspended in PBS with 3% BSA. Cells were stained with binder at indicated concentrations in PBS with 3% BSA for 1 hr at 4C. Cells were washed twice with PBS with 3% BSA. Cells were then incubated with ProtA-647 secondary (Thermo) at 1:1000 dilution in PBS with 3% BSA for 30 min at 4C. Cells were washed three times in PBS with 3% BSA cells and resuspended in PBS for flow cytometry analysis using a Beckman Coulter Cytoflex Flow Cytometer.

Instrument

Beckman Coulter Cytoflex Flow Cytometer

Software

FlowJo v10.8.1 software

Cell population abundance

Live vs. dead cells were gated using FSC and SSC area. Single cells were subsequently gated by doublet removal using FSC-H and FSC-A.

Gating strategy	Live vs. dead cells were gated using FSC and SSC area. Single cells were subsequently gated by doublet removal using FSC-H and FSC-A

☒ Tick this box to confirm that a figure exemplifying the gating strategy is provided in the Supplementary Information.
